# Management Accounting for Healthy Nutrition Education: Meta-Analysis

**DOI:** 10.3390/nu12123715

**Published:** 2020-12-01

**Authors:** Emilio Abad-Segura, Mariana-Daniela González-Zamar, José Gómez-Galán, César Bernal-Bravo

**Affiliations:** 1 Department of Economics and Business, University of Almeria, 04120 Almeria, Spain; 2 Department of Education, University of Almeria, 04120 Almeria, Spain; 3 Department of Education, University of Extremadura, Avda. de Elvas s/n, 06006 Badajoz, Spain; jgomez@unex.es; 4 Cupey Campus, College of Education, Ana G. Méndez University, San Juan, PR 00926, USA; 5 Department of Education Sciences, Language, Culture and Arts, Rey Juan Carlos University, Paseo Artilleros s/n, 28032 Madrid, Spain; cesar.bernal@urjc.es

**Keywords:** management accounting, nutrition education, healthcare cost, healthy, scientific production, meta-analysis

## Abstract

Unequal economic growth shapes food systems. Nutrition problems incorporate inappropriate practices, so nutrition education is key to empowering consumers to choose healthy foods. However, increasing the accessibility of healthy diets is related to reducing the cost of nutritious foods. The accounting management of healthy nutrition should allow for optimal global decision-making. The evolution of scientific production and global research trends on this topic between 1968 and 2019 have been studied. Statistical and mathematical methods have been applied to 1738 documents from the Scopus database. The results provided data on the agents that participate in the development of the theme. Data reveal an exponential trend, especially in the previous decade, with more than 50% of scientific production. Future lines of research have been identified: investment in health systems; green label education; early impact of food insecurity; WIC (Women, Infants, and Children) nutrition education; food waste audit; and ecological footprint of food. The central contribution of the study has been to detect the main future directions of research, providing critical points that will allow us to identify the themes of future publications, in addition to providing an instrument for decision-making carried out by the research funding sponsors.

## 1. Introduction

In 2019, two billion people globally did not have routine, regular access to safe, nutritious food in sufficient quantities [1]. This condition of food insecurity, in which people lack safe access to a sufficient amount of food for their development, health and daily activity, is mainly driven by the lack of food availability, lack of purchasing power, poor distribution and improper use of food [2,3].

Nutrition is constituted by the food which is consumed by an individual—that is, with the products of the agri-food sector. With the development of food systems (production, collection, storage, transportation, transformation and distribution of food), it is possible to improve people’s diet and health, in addition to decreasing the impact on natural resources. Nutrition problems generally include inappropriate practices [4,5,6]. Nutrition education is key to training consumers in making healthy food choices [7].

The precept of the Food and Agriculture Organization of the United Nations (FAO) is to eliminate hunger in the world by ensuring that countries have food security and good nutrition and cultivate sustainable agricultural methods. Consequently, the FAO points out that good nutrition is the first line of defense when fighting against diseases, as well as a source of energy for our daily activity [8].

Diets have hidden costs, which must be known in order to identify trade-offs and interactions in relation to the Sustainable Development Goals (SDGs) adopted by the United Nations (UN) in the 2030 Agenda for Sustainable Development [9]. The most critical hidden costs relate to the health (SDG 3) and climate (SDG 13) consequences of our dietary choices and the food systems that support them [10,11].

Likewise, the increased affordability of healthy diets is linked to the reduction in the cost of nutritious foods. The cost drivers (activity bases identified with the production or service process) of these diets are observed throughout the food supply chain, in the food environment and in the political economy that shapes trade policies, public spending and investment. Raising these cost drivers requires transformations in food systems without a global solution [12]. In order to reduce food losses and improve efficiency at all stages of the food supply chain, countries and territories need to rebalance agricultural policies, in addition to providing incentives for investments and more nutrition-sensitive political actions [13,14].

The purpose of this research is to detail the state of the knowledge base on the accounting management of health nutritional education, to identify the thematic axes considered and detect new directions. In the reviewed literature, studies have been found that pose and evaluate this question, so that the research questions on accounting management for a healthy nutritional education are:

(i) What is the development of scientific production in this field of knowledge?
(ii) What are the most prolific journals and main subject areas where the scientific articles of this research field are classified?
(iii) Which are the main authors, research institutions and territories/countries?
(iv) What are the disciplines or study areas that it develops?
(v) What are the future research directions that this topic addresses?

The aim of this research paper is to examine current and future lines of research at an international level on management accounting for healthy nutrition education, in the period 1968–2019—that is, from the first published article on the research topic (1968) to the last full year (2019). To obtain findings related to these research questions, a sample of 1738 scientific documents selected from the Scopus database has been analyzed.

The results obtained are valuable for the actors who continue to develop this scientific research theme of management accounting for healthy nutrition education and who require a study of past and future data, such as academics, scientific researchers, research institutions, higher education institutions, health personnel and suppliers of materials and services.

To accomplish the objective of this research, this work is structured as follows. Section 2 defines the unit of analysis and performs a review of the basic concepts or study variables. Section 3 describes the methodology used. Section 4 displays the results and discusses them in a broad research context. To finish, Section 5 summarizes and concludes.

## 2. Conceptual Framework

Section 2 is the outcome of a review of the literature, and its purpose is to represent a guide and framework for international research on management accounting for healthy nutrition education. The interrelation of the variables that conceptualize this field of knowledge is defined. The aim is to provide a conceptual framework that guides the research and enables the interpretation of the findings.

Table 1 displays the key research documents that focus on the theoretical and conceptual structure of the study topic. The analysis of these has allowed us to determine the questions, the purpose and the aim of the research, as well as to obtain the key terms (management accounting, nutrition education and healthy) to apply the methodology detailed in Section 3. For each of the articles, the following information is indicated: year of publication, title, author or authors, journal where they were published, thematic areas of the journal and the key terms that have been identified related to the objective of the research.

The reviewed literature offers definitions for the basic concepts of management accounting for healthy nutrition education. Some reflections of the concepts applied in the context of this research are incorporated, which have shaped this field. To avoid interpretations, the basic concepts of this subject have been defined and these will be used in the development of the study.

### 2.1. Management Accounting

Management accounting (or managerial accounting) is a modality based on the use of economic data obtained through other financial and cost accounting tools for subsequent decision-making in an organization. It is an accounting tool in constant evolution as an operational mix between financial and cost modalities [30]. Its main function is to carry out the final decision-making considering the most faithful possible image of the organization—that is, it looks at the immediate past to focus on the future. It involves the analysis of the organization’s structure and whether it corresponds efficiently with the objectives that it pursues [17,31].

The objectives of management accounting are planning (budgeting) and control, which require the anticipation of events, penetrating the temporary economic horizon. The preparation of plans and budgets requires the use of all suitable techniques to provide the most satisfactory solution possible to the problems that arise [32,33]. The inter-scientific collaboration in this area of management requires the contribution of economic theory, operational research, mathematics of financial operations, statistics, behavioral sciences and organization theories, among others. This interdisciplinary collaboration is increasingly evident and necessary given the globalization of markets, the growing uncertainty of the environment and the competitiveness of companies [28,34].

Likewise, management accounting refers to the instrument that, based on the information on the constitutive elements of costs, allows the elaboration and communication of timely and relevant information to facilitate the operational, tactical and strategic decisions of the company, serving as a basis for the planning and control of managerial actions [35,36].

The cost and affordability of healthy diets is a global challenge. Healthy diets are unaffordable for many people in all regions of the world, especially for those suffering from financial problems [37]. In relation to the dietary choices of people globally, the hidden costs and externalities associated with current food consumption patterns are inconvenient to health and the environment [38]. In this sense, proper accounting management focuses on designing methodologies and strategies that identify the drivers of the high cost of nutritious food and will offer policy and investment guidance for countries to transform their food systems and provide access to affordable healthy diets, in ways which promote trade-offs and contribute to maximum synergies for environmental sustainability [39,40].

### 2.2. Nutrition Education

Nutrition refers to the biological process in which animal and plant organisms absorb nutrients necessary for life from food. As a science, human nutrition investigates the relationship between food consumed by man and health, to maintain wellbeing and the preservation of human health. Good nutrition prevents many chronic diseases and is related to a healthy lifestyle [4,41].

The concept of food and nutrition education (FNE) refers to educational strategies designed to facilitate the voluntary adoption of behaviors related to food and nutrition that are conducive to health and wellbeing. It is a tool used to promote healthy eating in the population, which provides knowledge, information and training to people, empowers them and allows them to make informed decisions regarding their diet [42,43]. On the other hand, it can have a positive influence by modifying consumption, shopping, food preparation, attitudes and eating habits [44].

In the context of the realization of the human right to adequate food and the guarantee of food and nutritional security, indicated by the FAO, FNE is a field of permanent, transdisciplinary knowledge and practice, intersectoral and multiprofessional, which seeks to promote the autonomous and voluntary practice of healthy eating habits [5,45,46]. This human right is linked to the following Sustainable Development Goals (SDGs) adopted by UN in the 2030 Agenda for Sustainable Development: 1 (end of poverty) and 3 (zero hunger) [47,48]. The practice of the FNE should make use of problematic and active educational approaches and resources that favor dialogue with individuals and population groups, considering all stages of the life course [49].

FAO contributes to the concept of school-based food and nutrition education (SFNE), which consists of educational strategies and learning activities. Supported by a healthy eating environment, it facilitates students, adolescents and their communities to: (i) improve their diets and food choices; and (ii) develop their capacity to act as agents of change [50]. Additionally, the agency of the United Nations (UN) promotes a comprehensive school approach for SFNE, actively involving all people who interact in the educational environment (children, families, teachers, school personnel, local farmers, food service personnel, food sellers and government personnel) [5,51].

### 2.3. Health

The World Health Organization (WHO), the directing and coordinating authority in international health matters in the UN, in its constitution of 1948, indicated that health refers to “a state of complete physical well-being, mental and social, not only to the absence of disease or illness” [52].

Healthy eating consists of eating a variety of foods that provide the person with the necessary nutrients to stay healthy, feel good and have energy. These nutrients include proteins, carbohydrates, fats, water, vitamins and minerals [53,54]. Moreover, this diet covers all the nutritional needs in the stages of a person’s life; however, each individual has some nutritional requirements based on age, gender, height, physical activity and health or disease status [55,56,57].

A healthy diet must be: (i) complete, since it must provide all the nutrients the body needs; (ii) balanced, since the nutrients must be proportionate (carbohydrates—CHO: 55–60% of the total kcal per day; fats: 25–30%; and proteins: 12–15%), and include drinking 1.5–2 L of water a day; (iii) sufficient, since the amount of food must be adequate to keep the weight in the normal range and, in children, achieve proportional growth and development; (iv) adapted to age, sex, height, physical activity carried out, work carried out by the person and their state of health; (v) varied, since it will contain different foods from each of the groups (dairy, fruits, vegetables, cereals, legumes, meat and poultry or fish, among others) to guarantee all the necessary nutrients [58,59,60,61].

## 3. Methodology

Section 3 describes the bibliometric techniques applied in this study, the data inclusion and exclusion criteria to determine the final sample of articles analyzed and the data processing in relation to the research objective.

### 3.1. Method and Data Selection

Mathematical and statistical methods are applied to scientific literature to analyze the activity of a scientific theme. The explicit objective of this methodology is to search, identify, organize and analyze the trends of the research topic [62,63]. In recent decades, bibliometrics has contributed to the review of scientific knowledge and has been used successfully in different scientific fields [64,65,66]. The aim is to show a vision of the development of research related to management accounting for healthy nutrition education. For this, a quantitative analysis has been carried out applying mathematical and statistical techniques.

Nowadays, the advantages of bibliographic databases are undeniable since they provide the quality and validity of an analysis. Some studies have evaluated which database is most appropriate for use in bibliometric studies. Two scientific databases, Web of Science (WoS) and Scopus, raise the question about the comparison and stability of statistics obtained from different data sources.

The previous evaluations between both databases have not determined which is the best, since it all depends on the focus of the analysis, the discipline and the timeframe of the study [67]. In this study, both databases have been evaluated; therefore, the volume of documents in WoS (Core Collection) was 1139, while in Scopus, it was 1738, for the same time period.

**Figure 1:**
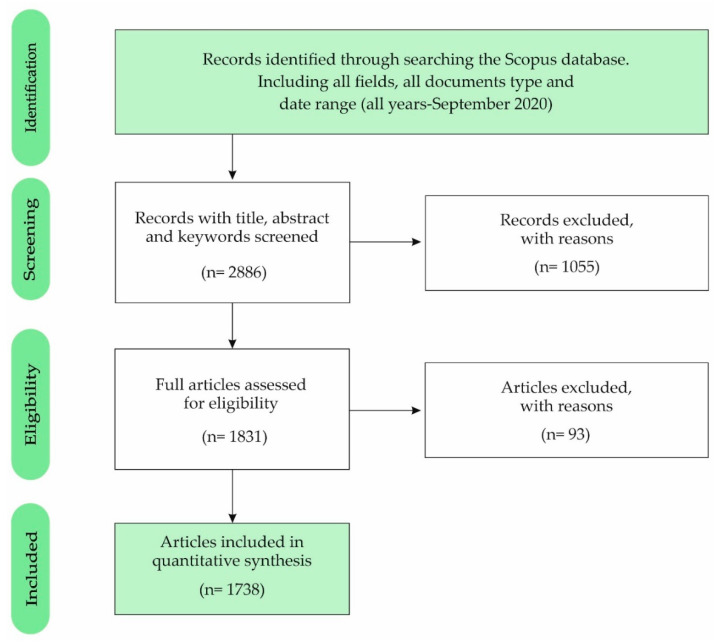
Sample of selected articles based on Preferred Reporting Items for Systematic Reviews and Meta-Analyses (PRISMA).

The process followed in the collection (inclusion–exclusion) of the sample conforms to the flow diagram of Figure 1, according to the Preferred Reporting Items for Systematic Reviews and Meta-Analyses (PRISMA) [68]. Phase 1 (Identification) detected 62,851 records from the Scopus database, considering, for each of the key search terms (cost, accounting, education, nutrient, food and health), “all fields”, “all types of documents” and “all the data published in the data range (all years-September, 2020). The search terms were identified in the first literature review (see the main documents reviewed in Table 1). In phase 2 (screening), the option “article title, abstract and keywords” was chosen in the field of each search term, so that 59,965 records were excluded. In phase 3 (eligibility), of the 2886 records, only “articles” were selected as the type of document, to ensure the quality derived from the peer review process. In this phase, 1055 records were excluded. Finally, in phase 4 (included), the data referring to the “all years-2019” period were selected—that is, from the first published article on the research topic (1968) to the last full year (2019). In this last phase, 93 of the 1831 records were excluded, so the final sample included 1738 articles, both open and non-open access.

The search selected documents from the subfields “title, abstract and keywords”, in the period that contains the publication of the first article on the research topic until the last full year (52 years), as has been applied successfully in other analyses [69,70,71]. The representativeness of the sample is based on the proven quality of the Scopus database in relation to the indexing protocol and with the systematic procedures of the search criteria.

### 3.2. Data Processing

Data processing takes place with the collection of data and its translation into usable information. This phase must be carried out correctly, in order not to affect the results obtained from the data. In this research, the variables analyzed were: (i) the year of publication of the articles; (ii) the subject area where each of the records is classified; (iii) the journal; (iv) the author; (vi) the research institution where the author is affiliated; (v) the author’s country of affiliation; and (vii) the keywords that define the publication.

Regarding bibliometric indicators, in this study, the following were used: (i) activity (quantity and quality)—they offer data on the volume and impact of research activities; and (ii) relationship (structural collaboration)—they offer track interactions [72]. The quality indicators used to measure the impact of the research were: (i) the number of citations, and (ii) the h-index, which refers to the maximum value h of a journal that has published h articles, cited at least h times [73].

Among the relationship indicators, the following analyses have been used: (i) co-citation: this is used when a document cites two others, displaying the probability that both sources cited are related by their content [74]; (ii) co-authorship: this studies the social structure of a research topic and is used to evaluate the patterns of scientific collaboration at the level of authors, institutions and countries, based on bibliographic data that provide information on the institutional affiliations of the authors and their geographic location [75]; and (iii) co-occurrence: this offers a graphical visualization of the relationships of the concepts represented in the papers and allows the detection of the main current and future research topics from the analysis of the keywords, since the documents can be reduced to the entirety of the joint appearances between the words that compose it [76]. The occurrence attribute reveals the number of documents in which a keyword appears.

In relation to the analysis of the relationship indicators, the VOSviewer software (v1.6.15, University of Leiden, Leiden, The Netherlands) has been used. This provides data on collaborations and the evaluation of the contents, to determine the activities of the networks’ research [77]. This tool allows us to recognize research trends based on the use of keywords in research articles.

### 3.3. Keyword and Term Co-Occurrence Analysis

In relation to the analysis of co-occurrences of terms of the scientific articles, and in order to identify the thematic axes (current and future) of the research topic, the terminology of VOSviewer has been used: (i) “link”, which refers to the links of co-occurrence between terms; (ii) “total link strength”, which refers to the strength (positive numerical value) of each link and, for concurrent links, this indicates the number of documents in which two terms appear together, (iii) “network map”, consisting of the set of terms and links; and (iv) “cluster”, which refers to the set of terms included in a network map; it is important to note that clusters do not need to comprehensively cover all the components of a network map [77,78].

The attributes used to describe the terms have been “weight” and “score”, denoted by numerical values. The “weight of a term” reveals the significance of the term in the field of research studied. For a term, the “link weight” reveals the number of links a term has with other terms, while the “link strength weight” indicates the total strength of the links of a term with other terms. The “score” attribute allows the keywords of the titles and summaries of the topic documents on management accounting for healthy nutrition education to be classified by relevance. Calculating the relevance score for each keyword assumes that the higher scoring terms provide a better prediction to identify a future line of research [79]. Hence, starting with the term *x* in research field *a*, which in turn is part of research field b, the relevance score of term *x* in research area *a* is calculated as follows (1):

(1) $$Relevance\;score\;\;=\;\;\frac{n_{ax}}{n_{bx}+c}\;,$$

where *n_ax_* and *n_bx_* are the number of elements in the areas *a* and *b* in which the term *x* appears, respectively.

This quotient is based on the equilibrium of (symbolized by parameter *c*) the frequency of appearance of *x* in area *a*, in relation to the frequency of appearance of *x* in area *b*, in addition to the absolute frequency of appearance of *x* in the area, which can be considered as an indication of the relevance of *x* to the area *a* [80,81].

## 4. Results and Discussion

### 4.1. Analysis of World Scientific Production

**Figure 2:**
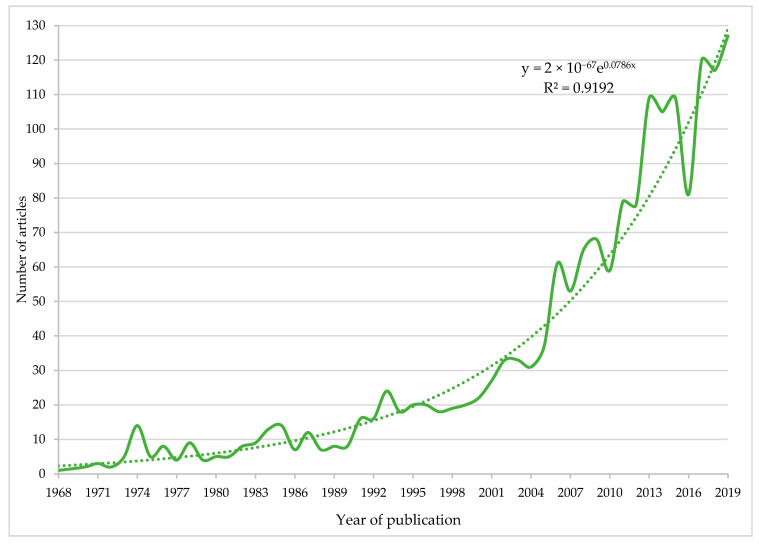
Exponential evolution of world scientific production (1968–2019). y: Ordinate axis-dependent variable (Number or articles); x: Abscissa axis-independent variable (Year); e: Euler’s number (2.7182) approximately; R^2^: Coefficient of determination.

Figure 2 displays the evolution of the number of articles published globally in the field of management accounting for healthy nutrition education research, in the period 1968–2019. An exponential trend in the publication of scientific documents is observed since the beginning of the period, more pronounced since 2000. Of the 1738 records published in the last 52 years analyzed, 554 were in the last 5 years (2015–2019), which represents 31.88%. The number accumulated in the last decade (2010–2019) was 984 articles (53.62%), while for the last 15 years, it was 1268 (72.96%). In 2019, 127 articles (7.31%) were published, the highest annual quantity of the period. These data confirm the interest in the subject in recent years by both the scientific and academic communities at a global level. The exponential trend line indicates the number of articles about this topic increasing faster over time. Furthermore, this line shows its goodness, with a coefficient of determination, R^2^, of 0.9192, referring to the proportion of the variance in the dependent variable (number of articles) that is predictable from the independent variable (year of publication).

Emerging challenges (climate change, environmental sustainability and technological transformation) are changing food systems and raising questions about how to sustainably feed a growing world population [82,83]. The FAO, in relation to its nutrition strategy, supports the actions derived from the Second International Conference on Nutrition (ICN2) of 2014, the UN Decade of Action on Nutrition (UNSCN) (2016–2025) by the General Assembly of the UN, and leads the implementation of its Work Program together with the WHO. This means a growth in research activity that develops guidelines on food-based nutrition, and socio-economic indicators based on food and human needs [84].

On this subject, 94.68% of the documents are published in English (1655). This circumstance is associated with the fact that publication in this language broadens a publication’s audience, as is the case widely in searches carried out in the Scopus database [85]. The other scientific articles have been published in other languages: Spanish (25, 1.43%), French (19, 1.09%), Chinese (10, 0.57%), German (9, 0.51%), Portuguese (7, 0.40%), Croatian and Italian (3, 0.17% each). The rest of the languages did not surpass 2% of the documents published.

### 4.2. Subject Areas and Journals

The 1738 records are classified into 26 subject areas in the Scopus database. The same article can be categorized into one or more than one thematic area. There is a positive correlation between the subject areas and the journals where the articles are published. The editor of the journal catalogues each article in subject areas, according to the criteria and experience.

**Figure 3:**
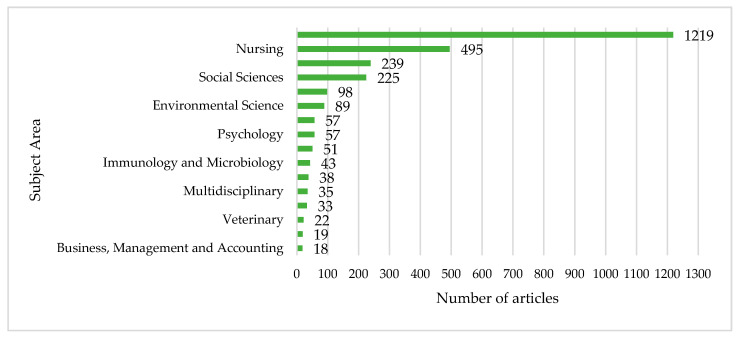
Main subject areas and number of articles associated (1968–2019). Note: the same article can be classified into one or more than one subject area.

Figure 3 shows the top subject areas where articles on management accounting for healthy nutrition education are classified. Medicine is the subject area that brings together the largest number of articles (1219 articles, 43.37%). It is followed by Nursing (495, 17.61%); Agricultural and Biological Sciences (239, 8.50%); Social Sciences (225, 8.00%); Biochemistry, Genetics, and Molecular Biology (98, 3.49%); Environmental Science (89, 3.17%); Pharmacology, Toxicology, and Pharmaceutics (57, 2.03%); and Psychology (57, 2.03%). The remaining subject areas constituted just 2% of the published articles.

The topic of management accounting for healthy nutrition education is a multidisciplinary research topic. Its analysis is multidimensional since its evolution reflects various disciplines [86]. However, in addition to its initial association with accounting, medicine and education, it is also linked by association with the fields of Psychology, Humanities, Economics, Neuroscience, or Biochemistry, Genetics and Molecular Biology [87,88].

In total, 1738 articles have been published in 915 international journals. According to the Scopus database, the 10 most productive journals, depending on the number of articles published on the subject, are: Journal of Nutrition Education and Behavior (63); Journal of the American Dietetic Association (53); Public Health Nutrition (41); BMC Public Health (32); PLOS ONE (25); Food and Nutrition Bulletin (22); Journal of the Academy of Nutrition and Dietetics (19); Appetite (18); Nutrients (16); and American Journal of Preventive Medicine (13).

**Figure 4:**
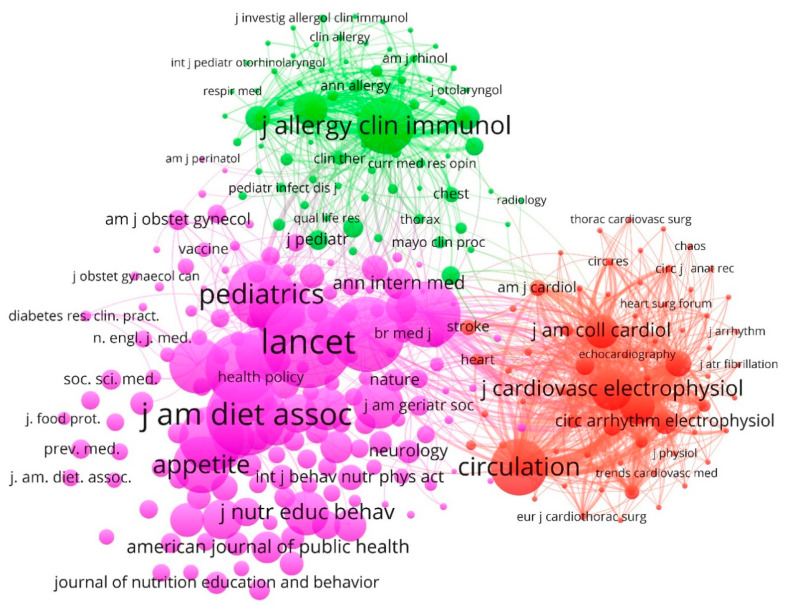
Network visualization of journals based on co-citation method (1968–2019).

Figure 4 shows the network map of the journals (abbreviated titles shown) that have been published around the world on management accounting for healthy nutrition education globally, based on co-citation analysis. The size of a journal’s circle is determined by the weight of the journal, so the higher its weight, the larger the circle. For some journals, the title cannot be displayed due to the density of the cluster and to avoid overlapping titles. The lines between the elements represent links. The color of a journal is determined by the cluster to which the journal belongs. The distance between two journals on the display map roughly indicates the relationship of the journals in terms of citation links. The scientific journals are connected by three groups. The network visualization, during the analysis period (1968–2019), shows a high concentration in the link of journals by co-citation.

Cluster 1 (pink color): This first group collects 53% of the journals and it is led by The Lancet, with a citations weight of 788. This journal is associated, among others, with Journal of the American Dietetic Association (638); JAMA: The Journal of the American Medical Association (571); The American Journal of Clinical Nutrition (520); Pediatrics (473); The New England Journal of Medicine (459); Public Health Nutrition (448); Appetite (363); The BMJ (353); and Diabetes Care (352).

Cluster 2 (green, 25%): This group is headed by The Journal of Allergy and Clinical Immunology (369) and it is associated, among others, with Allergy (153); Annals of Allergy, Asthma & Immunology (91); Clinical & Experimental Allergy (84); The Journal of Pediatrics (66); Medical Care (66); The American Journal of Medicine (56); Pediatric Allergy and Immunology (56); Chest (43); and Clinical Therapeutics (32).

Cluster 3 (red, 22%): This component is led by Circulation (361) and it is associated, among others, with Journal of Cardiovascular Electrophysiology (215); Heart Rhythm (159); Journal of the American College of Cardiology (153); EP Europace (100); Circulation: Arrhythmia and Electrophysiology (91); European Heart Journal (56); Pacing and Clinical Electrophysiology (53); The American Journal of Cardiology (46); and Stroke (42).

It is surprising that the peer-reviewed general medical journal, The Lancet, being the most cited and the leader of the largest group (cluster 1), is not in the top 10 most productive on this research topic. This circumstance may be due to the fact that none of the main specialized editions of the British journal focus specifically on the accounting aspect of nutritional education, although it does have the topics Adult: Education and Nutrition & Metabolism, which is why it stands out in this thematic [89].

Moreover, the first scientific article dates from 1968, written by Kerr, J.R., titled “Income and Expenditures: the Over-65 Age Group”. It was published in the Journal of Gerontology (publisher: Gerontological Society of America, Washington, DC, USA) linked to the subject area Biochemistry, Genetics, and Molecular Biology (Aging) [90].

The most cited article, with 1134 citations in October 2020, was written, in 2016, by Gaugler, J., James, B., Johnson, T., Scholz, K., and Weuve, J., titled “2016 Alzheimer’s disease facts and figures, and published by Alzheimer’s & Dementia” (publisher: Elsevier, Amsterdam, The Netherlands). The journal is linked to the following subject areas: Medicine (Health Policy, Geriatrics and Gerontology, Neurology (Clinical), Psychiatry and Mental Health) and Neuroscience (Developmental Neuroscience, Epidemiology and Cellular and Molecular Neuroscience) [91].

Regarding the number of citations, it is followed, with 828, by the article written in 1985, by Bernstein, M. J., with the title “Lowering Blood Cholesterol to Prevent Heart Disease”, and published by JAMA: The Journal of the American Medical Association (publisher: American Medical Association, Chicago, IL, USA). This journal is linked to the subject area of Medicine (General Medicine) [92].

The most relevant article—that is, the one that most closely matches the search terms in the Scopus database—was written in 2018, by Shaw, A.; Capetola, T.; Lawson, J.T.; Henderson-Wilson, C.; and Murphy, B., titled “The cost of sustainability in higher education: staff and student views of a campus food culture”. It was published in the International Journal of Sustainability in Higher Education (publisher: Emerald Publishing, Bingley, UK), linked to the subject area of Social Sciences (Education, and Human Factors and Ergonomics) [93].

### 4.3. Analysis of the Main Drivers

#### 4.3.1. Authors

The 1738 articles on this topic of study at an international level have been written by 6851 authors—that is, essentially an average of four researchers per article. The most productive author, with 10 contributions, was Drewnowski, A., affiliated with both the University of Washington and the Center for Public Health Nutrition, located in Seattle, United States. This author has been contributing to the subject for a decade (1968–2019), where his article entitled Poverty and obesity: The role of energy density and energy costs stands out, with 1527 citations, published in 2004 in the American Journal of Clinical Nutrition, together with Specter, S.E. (UCLA Health Sciences, Los Angeles, CA, USA).

**Figure 5:**
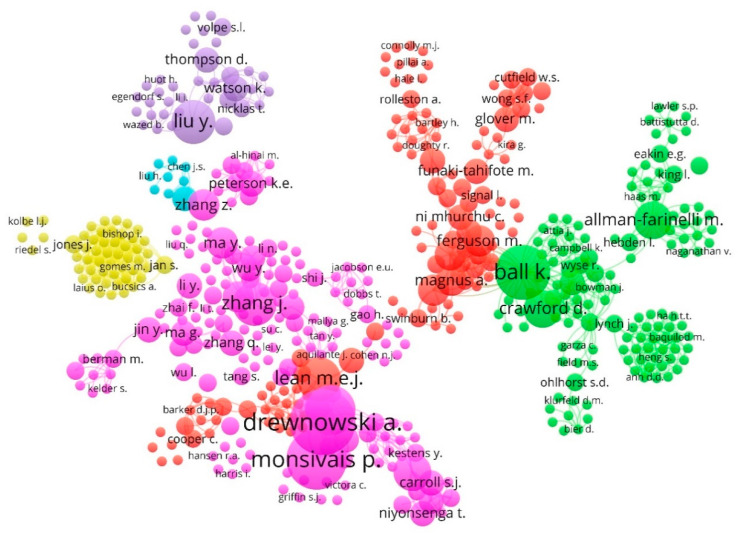
Network visualization of authors based on co-authorship method (1968–2019).

Figure 5 displays a collaboration map between the main authors based on the co-authorship analysis. The driving authors of this theme are associated in six clusters. The visualization network, during the period analyzed (1968–2019), shows a certain dispersion in the association of authors based on the co-authorship method.

Cluster 1 (pink): This is the largest and most central cluster, since it groups 33% of authors. It is the largest cluster, since it groups 20% of authors. It is led by Drewnowski, A. and he is associated, among others, with Monsivais, P.; Aggarwal, A.; Zhang, J.; Daniel, M.; Ma, Y; Wang, H.; Zhang, Z.; Carroll, S.J.; and Coffee, N.T.

Cluster 2 (green): The second groups 25% of the authors and it is headed by Ball, K. (Deakin University, Geelong, Australia). She is linked to Allman-Farinelli, M.; Crawford, D.; Campbell, K.J.; Campbell, E., Denney-Wilson, E., Eakin, E.G.; Hebden, L.; King, L.; and Lynch, J, among others.

Cluster 3 (red): This third group is made up of 23% of the authors and it is led by Lean, M.E.J. (University of Glasgow, Glasgow, UK). He is linked, among other authors, to Brimblecombe, J.; Ferguson, M.; Magnus, A.; Moodie, M.; Anderson, A.S.; Funaki-Tahifote, M.; Glover, M.; Jiang, Y.; and Mhurchu, C.N.

Cluster 4 (yellow): This fourth group groups 10% and it is led by Jan, S. (Rutgers University–New Brunswick, New Brunswick, NJ, USA). She is associated with Jones, J.; Baumgärte, C.; Bennie, M.; Bishop, I.; Brzezinska, A.; Bucsics, A.; Campbell, S.; Diogene, E.; and Ferrario, A, among other authors.

Cluster 5 (purple): This fifth group groups 7% and it is led by Liu, Y. (Tufts University, Medford, United States). She is liked to Thompson, D.; Watson, K.; Baranowski, T.; Cullen, K.; Jahns, L.; Nicklas, T.; Volpe, S.L.; Baranowski, J.; and Bhatt, R., among others.

Cluster 6 (cyan): This is the smallest cluster since it only constitutes 2%. It is led by Sun, J. (Mayo Foundation for Medical Education and Research, Rochester, New York, USA) and he is associated with Chen, J.S.; Ding, G.Q.; Huo, J.S.; Liu, H.; Lu, Z.X.; Luo, J.B.; Wei, Y.L.; and Zhang, R.H.

The author of Polish origin and affiliated with American institutions, Adam Drewnowski, heads cluster 1 (the central and most numerous) and is Professor of Epidemiology at the University of Washington and Director of the Center for Nutrition for Public Health at the School of Public Health of the University. His interest in public health, obesity and nutrition allows him to direct his contributions to the education, health and accounting aspects of this subject [94,95].

**Figure 6:**
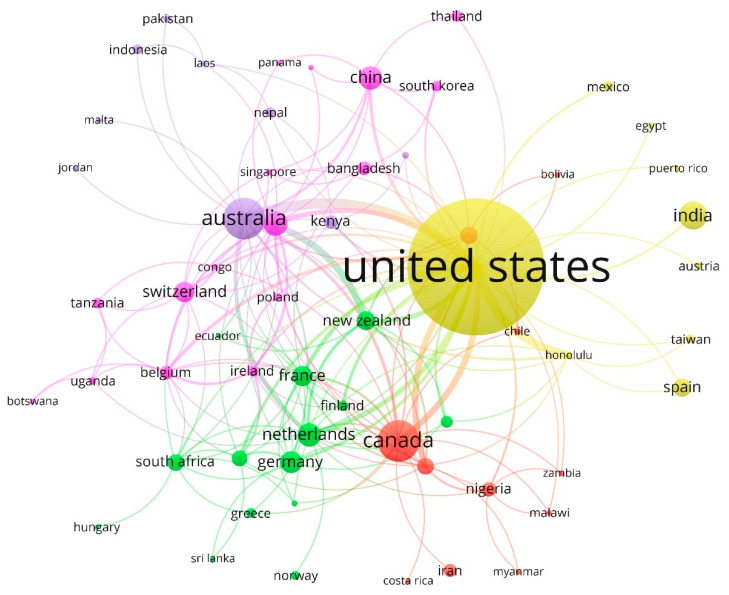
Network visualization of countries based on co-authorship method (1968–2019).

It is also noteworthy that of the six groups, four are led by authors affiliated with American institutions (Drewnowski, A.; Jan, S.; Liu, Y.; and Sun, J.); on the other hand, this result is related to the fact that the United States is the most productive and collaborative country in this field (see Section 4.3.3 and Figure 6 below) [96].

#### 4.3.2. Research Institutions

The 1738 articles on the research topic have been developed by authors affiliated with 4596 different international research institutions. Table 2 shows the 10 most productive research institutions on this topic during the 1968–2019 period. This ranking highlights that nine are North American: eight from the United States (The University of North Carolina at Chapel Hill (Chapel Hill, NC, USA), Harvard Medical School (Boston, MA, USA), University of Washington (Seattle, WA, USA), University of California (Los Angeles, CA, USA), Centers for Disease Control and Prevention (CDC) (Atlanta, GA, USA), United States Department of Agriculture (Washington, DC, USA), Deakin University (Victoria, Australia) and Harvard T.H. Chan School of Public Health (Boston, MA, USA)) and one Canadian (University of Toronto, Toronto, ON, Canada); and one is of Australian origin (The University of Sydney, Sydney, Australia).

The 10 most productive research institutions classify their articles in the thematic area of Medicine, followed by Nursing, in nine. This is related in the same way with the classification of all the articles in the sample (see Figure 3). The American institution, The University of North Carolina (Chapel Hill, NC, USA) at Chapel Hill, is the most productive, with 31 articles. It is also the one with the highest h-index (15). Of this group of institutions, the CDC, with 21 articles published, is the one that has been contributing to this topic for the longest period (1974–2019), followed by the University of California, with 22 articles, during the period 1977–2019. The 10 institutions in this table have published in the last year analyzed, 2019, indicating interest in this topic at an international level.

Furthermore, Table 2 shows the three main keywords of the articles published by the authors affiliated with the most productive research institutions on this topic. The most used keyword is “Health Care Cost”, used by four institutions (University of Toronto (Toronto, ON, Canada), Harvard Medical School (Boston, MA, USA), CDC (Boston, MA, USA) and Harvard TH Chan School of Public Health (Boston, MA, USA)), highlighting the accounting aspect of this thematic. They are followed, in order of use, by “Cost Effectiveness Analysis” and “Feeding Behavior”, by three institutions each; and “Food Insecurity” and “Health Behavior”, by three institutions.

It is noteworthy that the main authors direct their contributions towards analyzing the economic–accounting aspect of nutrition, as indicated by the most used keywords, corroborating the importance of managing resources to optimize the educational approach to nutrition at a global level [97,98].

#### 4.3.3. Countries

In the 52 years examined, 1738 records have been published by 118 countries/territories. The United States was the most productive country, with 798 published articles, which represents 45.91% of the total. It was followed by the United Kingdom (170, 9.78%), Australia (110, 6.33%), Canada (108, 6.21%) and India (58, 3.34%). The rest of the countries/territories did not reach 50 articles published during the time horizon examined.

Figure 6 shows the visualization network based on co-authorship analysis of the countries/territories driving this research topic. These are associated in five clusters. The network map, during the period analyzed (1968–2019), shows a certain dispersion in the association of countries/territories based on the co-authorship method.

Cluster 1 (pink): This conglomerate groups 27% of the countries/territories. It is led by Italy, with a link weight of 15 (links between countries). This is associated, among others, with Belgium (12), Switzerland (10), China (9), Ireland (9), Congo (6), Bangladesh (5), Uganda (5), Singapore (5), Tanzania (4), South Korea (3) and Poland (3).

Cluster 2 (green, 24%): This group is headed by the Netherlands (14) and is mainly associated with France (14), South Africa (12), Germany (11), Denmark (9), New Zealand (8), Finland (6), Japan (5), Portugal (4), Greece (3) and Ecuador (3).

Cluster 3 (red, 19%): The third set is headed by Canada (16) and is mainly associated with Sweden (13), Brazil (8), Nigeria (7), Chile (3), Malawi (3), Zambia (3), Myanmar (2), Bolivia (2) and Iran (1).

Cluster 4 (yellow, 15%): The fourth group is led by the United States (41), the most productive country in this research topic, and is weakly linked, among others, with Honolulu (8), Spain (2), Taiwan (2), India (1), Mexico (1), Austria (1), Egypt (1) and Puerto Rico (1).

Cluster 5 (purple, 15%): The fifth group, the least numerous along with the fourth, is headed by the most productive country in this research topic, Australia (21), and is associated with Kenya (6), Laos (5), Nepal (4), Indonesia (2), Pakistan (2), Ghana (2), Jordan (1) and Malta (1).

The United States stands out for its research potential in nutritional terms since they consider obesity a national problem and make efforts to regulate it. Derivative diseases (high blood pressure, heart disease, joint problems, osteoarthritis, apnea and other respiratory conditions, cancer, metabolic syndrome and, above all, type 2 diabetes) generate high medical costs. Consequently, to improve regulation, food chains have been encouraged to display caloric information in a format that is more understandable to the average consumer (minutes of walking, or kilometers of running, to burn the energy of a menu item) [25,99,100]. Likewise, as shown in Figure 6, their research links with countries such as Canada or Australia stand out.

### 4.4. Analysis of Keywords: Detection of Current Lines of Research

In the sample of 1738 scientific articles on management accounting for healthy nutritional education, 10,588 keywords have been identified. The 15 most prominent terms in the analyzed sample are “health education” (in 397 articles), “economics” (344), “diet” (308), “middle aged” (304), “health care cost” (304), “adolescent” (276), “nutrition” (276) “health promotion” (264), “feeding behavior” (223), “catering service” (214), “attitude to health” (197), “obesity” (197), “socioeconomics” (191), “food intake” (185) and “health care policy” (174).

These main terms are linked to the search terms or research variables identified in the initial literature review (see Table 1). Thereby, the variable “management accounting” is linked to health care cost or catering service; “nutrition education” to health education, diet, adolescent, feeding behavior, obesity or food intake; and “health” to health promotion or attitude to health.

**Figure 7:**
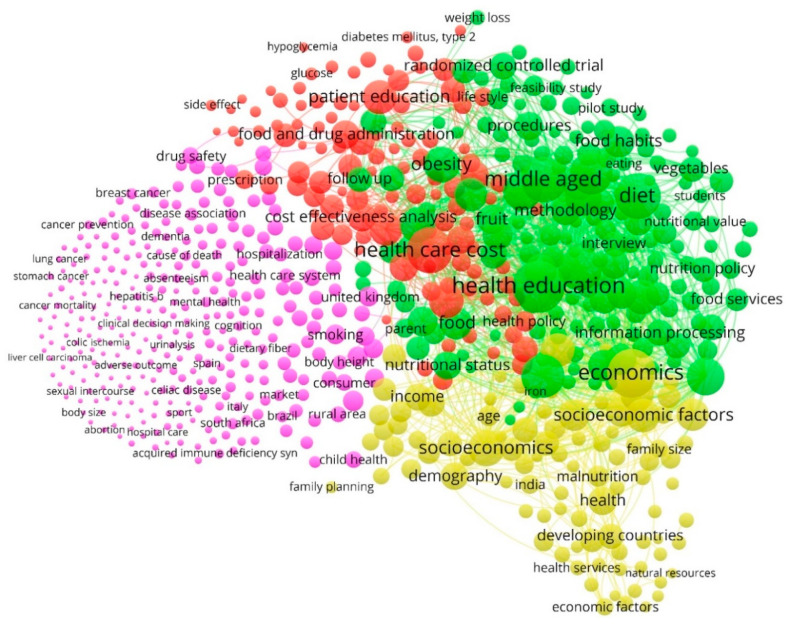
Network of keywords based on co-occurrence method (1968–2019).

A co-occurrence analysis of the keywords in the 1738 articles selected from the Scopus database was carried out. Figure 7 represents the network visualization of the identified keywords from the sample of articles on the study topic. The analysis of co-occurrences has made it possible to detect that the keywords were grouped into four well-differentiated clusters, identifying each cluster with a color. Cluster 1 is the most numerous, but its keywords are the ones with the fewest occurrences. Clusters 2 and 3 are the most central, which is reflected by the keywords with the highest link strengths. Finally, cluster 4 is the one that contains the least number of terms, although they are more represented in the articles than those of cluster 1. On the other hand, this analysis allowed us to recognize the lines of research developed by the main driving agents of this research field and based on the use of keywords in the articles, during the period 1968–2019.

Table 3 shows the four clusters detected, the color with which each cluster is identified and the weight that each group represents over the total sample. For each cluster, ordered by the number of occurrences, the main keyword is shown, which defines its name, in addition to the six most prominent keywords with which it is associated within the same component. Therefore, the lines of research identified were: “Diet Supplementation”, “Health Education”, “Health Care Cost” and “Economics”. In turn, for each keyword, the occurrences, the links and the total link strength are provided.

Cluster 1—Diet Supplementation

This is represented by the concepts of smoking, sex difference, health status, family, chronic disease and Internet. This line of research examines substances that can be used to add nutrients to the diet or to reduce the risk of developing health problems, such as osteoporosis or arthritis. Analysis of this school of thought indicates that dietary supplements must contain vitamins, minerals, fiber, amino acids, herbs or other plants or enzymes. Moreover, it has revealed that certain supplements can help to ensure that the individual receives enough vital substances that the body needs to function, or they help to reduce the risk of disease, although it emphasizes that supplements should not completely replace foods that are necessary for a healthy diet, as indicated by the Food and Drug Administration (FDA), federal agency of the Department of Health and Human Services (HHS) of the United States [101,102].

Cluster 2—Health Education

This thematic axis is represented, among other keywords, by diet, middle age, adolescence, nutrition, feeding behavior and attitude to health. During the timeframe examined, this line has established that health education refers to the process of education and participation of the individual, patient or family, to acquire the necessary knowledge, attitudes and basic habits to ensure the promotion and defense of the individual and collective health. Likewise, the research has been developed in parallel with the principles of the United Nations Educational, Scientific and Cultural Organization (UNESCO), which indicates that quality education is the foundation of health and wellbeing. That is, to lead a productive and healthy life, each person must have the necessary knowledge for the prevention of diseases and pathologies [103,104].

Cluster 3—Health Care Cost

This line of research is supported by terms such as health care policy, patient education, risk factor, cost effectiveness analysis, food and drug administration or prevalence [105]. Hence, this axis focuses on exposing the fundamental aspects of hospital costs to develop cost information systems and hospital management. The aim of this line has been to determine the optimization of hospital resources from an economic–social perspective, through cost-minimization, cost-benefit, cost-effectiveness and cost-utility analysis [106,107].

Cluster 4—Economics

This fourth line of research is represented by terms such as socioeconomics, public health, poverty, organization and management, income, and health care delivery. It has focused on analyzing the relationship between the socioeconomic status of the individual or of a population, education and healthy eating. The evidence highlights that among the economic factors related to nutritional education are the purchasing power of the individual; the consumption of less healthy food or diet patterns in people with a lower socioeconomic level; lower accessibility to the purchase of healthy foods in areas with a lower socioeconomic level; or the number of fast food establishments in urban areas. This thematic axis has identified socio-political strategies to favor accessibility to healthy foods and reduce costs [108,109,110].

### 4.5. Evolution of Keywords

**Figure 8:**
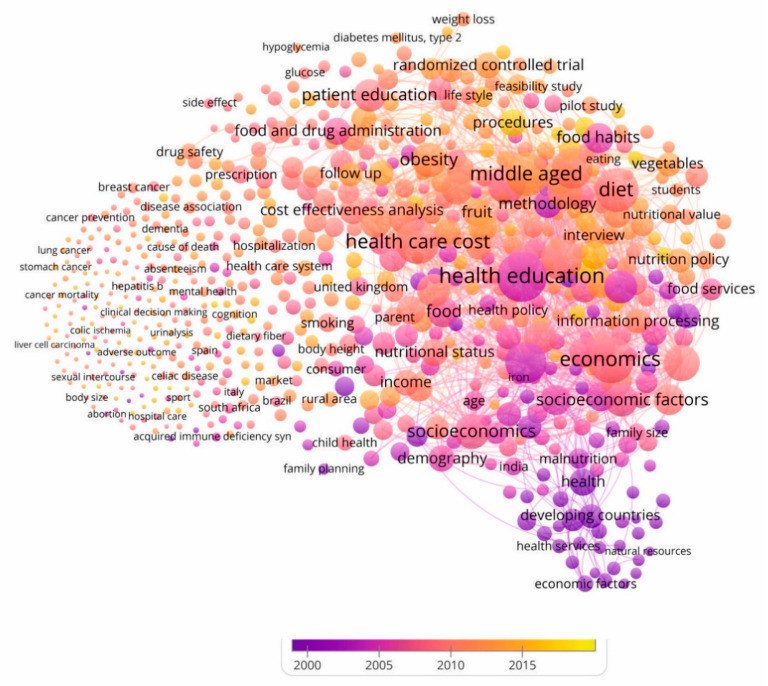
Association of keywords to clusters throughout the period (1968–2019).

Figure 8 displays and visualizes the evolution of the keywords of each of the four detected, by distinguishing the period in which they were incorporated by the driving agents of this topic. This allows us to understand the importance of keywords according to the time in which they have appeared, since the former have been a reference for the later ones, as the lines of research were developed.

For this reason, the existence of four well-differentiated clusters (see also Figure 7) allows us to understand how research on management accounting for healthy nutritional education has been growing.

In this evolution of keywords, Figure 8 shows that the coalescence of the group of pioneering keywords (purple color) made it possible to form the basis of the study. This group includes keywords such as dietetics, diseases, family food habit, fast food culture, food addiction, health care delivery, health services, health, nutrition health education, nutrition insecurity, nutritional disorders, nutritional needs or prevention. These first contributions developed the basic concepts that would support the research field on food and nutrition education from the perspective of the prevention and control of malnutrition problems and chronic diseases related to diet [111].

In the central group were the terms cost of illness, eating habit, effective nutrition communication, food preference, genetic predisposition, health care facility, health care management, health policy, health program, income, lowest income group, medical education, Mediterranean diet, mental disease, micronutrients, nutritional health, patient education, quality of life, social impact accounting or substance abuse. Subsequently, the works were directed towards research into the problems that cause malnutrition, vitamin and mineral deficiency and obesity in many countries, along with chronic diseases related to excessive or unbalanced diets [18,112].

Meanwhile, the keywords that have been incorporated more recently include antibiotic therapy, body fat, cardiometabolic risk, consumer attitude, ecological footprint accounting, food insecurity, healthy eating index, nutrition science, optimal nutrition, organic food, risk reduction behavior, social norm or soft drink. Later, contributions to this topic have been focused on establishing the requirements that must be met in order for the food that is put on the market to be considered safe, without losing sight of the fact that this safety reaches consumers with special nutritional needs [113]. In the same way, the works have analyzed the effects of putting unsafe products on the market by economic operators, to examine the obligation to collaborate with the administrations and proceed to withdraw the products from the market when the safety of the products is not guaranteed. Food safety and nutrition subjects are multidisciplinary subjects and there are disciplines and regulations that address specific aspects of the subject of accounting management in nutritional education [17,25,114].

The different sub-periods in which scientific activity on this topic has developed during 1968–2019 represent an abundant collection of key terms. In the titles, abstracts and keywords of the articles in the sample, VOSviewer has identified 10,588 different keywords. This makes it possible to validate the breadth of study axes in research activity.

### 4.6. Suggestions for Future Research

After reviewing the literature on management accounting for healthy nutritional education, along with a detailed examination of the latest analyses being carried out by the main drivers of this topic and an analysis of the keywords and their current trends, we identified the different future directions that this field of research may develop. International research in this field continues to evolve while incorporating concepts that establish new approaches that propose new lines of research. The set of the latest terms associated with this research has been identified, which has made it possible to link them to new directions in the research.

The clustering analysis consisted of separating the analysis units into groups of similar elements and then determining the most novel terms from the relevance score (see Section 3.3). The terms achieved would be assimilable to future thematic lines in this field of research. This is an effective process for discovering trends and emerging issues in a scientific discipline. Table 4 displays the future research directions detected by the relevance score and the main associated terms that provide a more defined approach to each of them. Subsequently, a description of each research direction identified is provided.

#### 4.6.1. Quantifying Health Systems Investment

Analysis of investment in health systems at a global level, with the basic aim of strengthening capacities in terms of workers, critical medical products and infrastructures to save lives. The COVID-19 pandemic has revealed existing structural problems, such as those arising from deficiencies in investment in physical infrastructure, as well as in the hiring and working conditions of health personnel. The research should focus on the examination of strategies that improve the resilience of the health system, reducing regional disparities in terms of spending, physical resources and personnel. In this sense, the European Commission seeks to guarantee continuous access to medical and social care and, in the medium term, the decrease in resources due to the economic crisis derived from the pandemic should not translate into a fall in investment in healthcare [115]. It is key to prioritize financing in education, employment and leadership of health personnel. Likewise, the WHO recognizes the value of a robust health system to achieve global goals related to universal health coverage, mental health and non-communicable diseases, emergency preparedness and response, security of the patient and the provision of integrated person-centered care [116].

Contributions should study the effect of investment in primary prevention in terms of nutrition, with an approach that emphasizes lifestyle behavior modification. For example, in relation to obesity, studies should examine how countries have effectively employed public awareness campaigns, training of health professionals and advocacy for dietary change and the sustainable effects of putting limits on unhealthy foods, taxes and labeling nutrition, as noted by the Global Future Council on Health and Healthcare (World Economic Forum, 2020) [117].

#### 4.6.2. Green Label Education

Examine and update the approach, initiated at the UNESCO, referred to as the United Nations Environment Programme (UNEP or UN Environment) International Congress on Education and Training on the Environment (Moscow, 1987), which supported environmental education as a permanent course in which individuals and communities become aware of their environment and learn the knowledge, ethics, skills, experience and the determination that enables them to act, individually and collectively, in the resolution of present and future environmental complications [118]. Nowadays, there is a current of authors who state that, in education, there must be a green pedagogy, which establishes that each person is a seed that has everything they need to develop within. Education requires feedback processes, where the values that are promoted are equality, respect, empathy, dignity and cooperation [119,120].

Diverse organizations have gathered around the Platform for the Performance of Sustainability in Education to create tools to evaluate sustainability in universities and colleges globally. The French government, non-governmental organizations and the French university associations (Conférence des Grandes Ecoles and Conference of University Presidents) elaborated the framework of the Green Plan as a strategy for sustainable development within the framework of the Grenelle Environment Roundtable, of the European Strategy for Sustainable Development and ISO 26000-Social Responsibility [121]. This Green Plan was created to indicate the aims of each establishment and the points that can be progressively implemented according to their pace, status, associations and individual situation. This tool allows the evaluation of the progress of the sustainable development actions implemented in the institution (self-diagnosis, scorecard, strategy guide and basis for certification). It has been conceived as an initial step to obtain a green label in relation to the activities developed by these institutions: strategy and governance, social policy and territorial presence, environmental management, teaching and training or research activities [122].

#### 4.6.3. Early Food Insecurity Impact

This thematic axis must develop the different factors inherent to food insecurity for its early detection. This concept refers to the situation where people lack safe access to enough food for their development and lead an active and healthy life. Moreover, food insecurity results from numerous reasons, such as the lack of availability of food, the lack of purchasing power, the poor distribution of food or the inappropriate use of it [123]. Due to these factors, early detection of lack of access to food is key to improving people’s lives and reducing the number of undernourished. Food insecurity (chronic, seasonal and transitory), as indicated by the Office of Disease Prevention and Health Promotion (ODPHP), allows the classification of citizens who are in this state, to promote the most appropriate measures for the elimination of this problem [124].

#### 4.6.4. WIC (Women, Infants and Children) Nutrition Education

This line should develop scientific evidence about pregnancy, childhood and early childhood as periods of rapid growth and physiological development. In this sense, insufficient nutrition during these periods of development places babies and children at risk of reduced emotional and cognitive development, in addition to negative health outcomes [125,126].

Many programs educate pregnant and postpartum women about the importance of good nutrition and advise them to feed healthy and nutritious food to their children and families. The Special Supplemental Nutrition Program for Women, Infants, and Children (WIC) offers supplemental foods, nutrition education and health and social services for pregnant, lactating and postpartum women and children aged one to four at nutritional risk [127]. The purpose of this program is to provide good medical care during critical developmental periods to prevent the onset of health complications. To accomplish this, it offers complementary foods, nutrition education and recommendations from healthcare and social service providers.

#### 4.6.5. Food Waste Audit

Among the main objectives of the fight against food waste are to change the behavior of consumers, to adopt an efficient attitude and to overcome the current mentality of throwing away. Food waste audits involve and bring together diverse stakeholders. Based on data collection, audits determine which foods are not eaten and why, within a specified period. Moreover, these audits help to inform people about the amount of food they waste and stimulate the consumption of more nutritious foods to reduce food waste [128,129]. Once the products that are not consumed have been identified, strategies are implemented to reduce food waste, in addition to advising on the reduction of food waste and favoring the consumption of all foods, as UNEP points out [130].

#### 4.6.6. Ecological Footprint of Food

Assessment of the impact of the consumption of products/services of any kind in nature, and its measurement based on the ecological footprint. This is an environmental indicator of the impact that a community, country, region or city has on its surroundings [131,132]. For food, the ecological footprint is expressed as the area necessary to produce the resources consumed by any activity, in addition to that necessary to absorb the waste it generates, regardless of its location [133]. The ecological footprint of a food is calculated from the surfaces, aquatic and terrestrial, necessary to produce animal or vegetable food (crops, grazing and fishing), including the energy costs associated with its production. Currently, the ecological footprint is 2.8 global hectares per inhabitant (Global Footprint Network, 2018) [134], which indicates that two planets Earth would be needed to satisfy current consumption needs and the generation of resources.

## 5. Conclusions

The objective of this study was to analyze the evolution of scientific production and research trends at a global level, during the last 52 years (1968–2019), on management accounting for a healthy nutritional education.

Of the 26 thematic areas in which the publications are classified, Medicine is the one that brings together the largest number (43.34%), followed by Nursing (17.61%). Four main thematic axes developed by the promoters of this subject during the period examined have been detected: (i) diet supplementation; (ii) health education; (iii) health care cost; and (iv) economics.

Internationally, research on management accounting for healthy nutritional education continues to evolve, so six future directions for research have been identified: (i) health systems investment; (ii) green label education; (iii) early food insecurity impact; (iv) WIC (women, infants and children) nutrition education; (v) food waste audit; and (vi) ecological footprint of food.

The different schools of thought in this field of study have been identified from the collaboration of groups of authors, institutions, countries/territories, journals and keywords. The results obtained are a complement to the knowledge of this subject and allow us to base the decision-making process in relation to nutritional education at all levels that form healthiness. With appropriate nutritional education, the quality of life of citizens would benefit, so that the pressure on health systems would be gradually eliminated, and institutions would be allowed to achieve an optimized management of resources at a lower cost.

The research helps to generate new qualitative knowledge and serves as a prelude to future discussions by presenting a broad vision of the landscapes of the research developed and from which lines of emerging interest are identified. These future directions in research allow academics/researchers to direct their next publications and health/educational institutions and funding entities to guarantee an adequate approach to their objectives.

The development of this study has a set of limitations, which have obviously conditioned the results obtained. These circumstances may be the basis for future contributions in this field of research that would provide different approaches. Among the limitations are: (i) the Scopus database chosen to apply the methodology; (ii) the keywords selected to extract the article sample; (iii) the time horizon of the study; (iv) the bibliometric techniques used; (v) the variables examined; (vi) the software used to obtain the network maps; or (vii) the English language of the articles examined. It is necessary to emphasize in this sense that using data mining techniques, large databases can be examined and patterns that explain their behavior can be found.

Finally, it has been observed that international research on the accounting management of healthy nutritional education presents an exponential trend, derived from both the number of articles and current and future lines of research, which indicates the growing interest in the academic/scientific community to find solutions to the problems that arise in a society in constant progress. In other words, scientific activity is developing in an optimal environment, and with a global interest in the dissemination of publications, thus allowing progress in this field of study.

## Figures and Tables

**Table 1:** Key articles reviewed to establish the research aim.

Year	Title (Reference)	Author	Journal	Subject Area	Key Term
2020	Health Inequities and the Shifting Paradigms of Food Security, Food Insecurity, and Food Sovereignty [15]	Borras, A.M.; Mohamed, F.A.	*International Journal of Health Services*	1	NE-H
2020	Prevalence of Product Claims and Marketing Buzzwords Found on Health Food Snack Products Does Not Relate to Nutrient Profile [16]	Breen, M.; James, H.; Rangan, A.; Gemming, L.	*Nutrients*	2–3	NE-H
2020	Needs and cost-effectiveness in health care priority setting [17]	Gustavsson, E.; Tinghög, G.	*Health and Technology*	4–5–6–7	MA-H
2019	Accounting for the Nutritional Context to Correctly Interpret Results from Studies of Exercise and Sedentary Behavior [18]	Braun, B.; Newman, A.	*Nutrients*	2–3	MA-NE
2012	An economic perspective on childhood obesity: Recent findings on cost of illness and cost effectiveness of interventions [19]	John, J.; Wolfenstetter, S. B.; Wenig, C.M.	*Nutrition*	1–3	MA-NE-H
2011	What is the real cost of our food? Implications for the environment, society and public health nutrition [20]	O’Kane, G.	*Public Health Nutrition*	1–3	MA-NE-H
2009	Why Does My Food Suddenly Cost So Much? [21]	Tillotson, J.	*Nutrition Today*	3	MA-NE
2004	Do Healthier Diets Cost More? [22]	Drewnowski, A.; Barratt-Fornell, A.	*Nutrition Today*	3	MA-NE
2002	A Cost Constraint Alone Has Adverse Effects on Food Selection and Nutrient Density: An Analysis of Human Diets by Linear Programming [23]	Darmon, N.; Ferguson, E.L.; Briend, A.	*The Journal of Nutrition*	1–3	MA-NE
2002	Preventive nutrition: consideration of cost-benefit and cost-effective ratios [24]	Chandra, R.K.	*Nutrition Research*	1–3–4	MA-NE
1997	Workshop on cost-effectiveness in health and medicine [25]	Glade, M.J.	*Nutrition*	1–3	MA-H
1996	Case Management in Home Total Parenteral Nutrition: A Cost-Identification Analysis [26]	Curtas, S.; Hariri, R.; Steiger, E.	*Journal of Parenteral and Enteral Nutrition*	1–3	MA-NE
1992	Improving the Cost Effectiveness of Nutrition Education [27]	McNutt, K.	*Nutrition Today*	3	MA-NE
1986	Cost-effectiveness and cost-benefit analysis of worksite nutrition programs [28]	Joseph, H.M.; Glanz, K.	*Journal of Nutrition Education*	1	MA-NE
1982	Food shortage simulations: An application of the food accounting matrix with proportionality assumptions [29]	Freeman, R.; Hay, R.	*Ecology of Food and Nutrition*	1–2–8	MA-NE

1: Medicine; 2: Agricultural and Biological Sciences; 3: Nursing; 4: Biochemistry, Genetics, and Molecular Biology; 5: Immunology and Microbiology; 6: Chemical Engineering; 7: Engineering; 8: Environmental Science; MA: Management Accounting; NE: Nutrition Education; H: Healthy.

**Table 2:** Top 10 research institutions (1968–2019).

Research Institution	A	Country	Subject Area	h *	1A *	LA *	Keyword 1	Keyword 2	Keyword 3
The University of North Carolina at Chapel Hill	31	USA	ME-N-P	15	1986	2019	Health Promotion	Feeding Behavior	Health Behavior
University of Toronto	26	Canada	ME-BGM-N	11	1986	2019	Health Care Cost	Health Care Policy	Social Status
Harvard Medical School	22	USA	ME-BGM-A	13	1974	2019	Health Care Cost	Food Insecurity	Cost Effectiveness Analysis
University of Washington	22	USA	ME-N-SS	11	1992	2019	Socioeconomic Factors	Educational Status	Caloric Intake
University of California	22	USA	ME-N-AB	11	1977	2019	Medical Education	Food Insecurity	Physical Activity
Centers for Disease Control and Prevention (CDC)	21	USA	ME-SS-N	13	1974	2019	Health Care Cost	Public Health	Cost Effectiveness Analysis
United States Department of Agriculture (USDA)	20	USA	AB-N-ME	13	1983	2019	Feeding Behavior	Food Habits	Food Quality
Deakin University	19	USA	ME-N-P	11	2005	2019	Cost Benefit Analysis	Food Preferences	Health Behavior
The University of Sydney	19	Australia	ME-N-AB	12	2004	2019	Diet	Feeding Behavior	Obesity
Harvard T.H. Chan School of Public Health	19	USA	ME-N-AB	11	1993	2019	Health Care Cost	Cardiovascular Disease	Cost Effectiveness Analysis

A: number of articles; ME: Medicine; SS: Social Sciences; N: Nursing; P: Psychology; BGM: Biochemistry, Genetics and Molecular Biology; AB: Agricultural and Biological Sciences; h: h-index or Hirsch index; 1A: First article; LA: Last article; (*): in this research topic.

**Table 3:** Cluster and main associated terms (1968–2019).

Cluster Number	Cluster Color (See in Figure 7)	%	Keywords	Occurrences	Links	Link Strength
1	Pink	46%	Diet Supplementation *	58	599	1470
			Smoking	58	579	1449
			Sex Difference	57	589	1614
			Health Status	55	599	1411
			Family	52	601	1409
			Chronic Disease	50	586	1397
			Internet	50	581	1238
2	Green	24%	Health Education *	397	588	6695
			Diet	308	510	5663
			Middle Aged	304	544	5649
			Adolescent	276	546	5041
			Nutrition	276	660	5180
			Feeding Behavior	223	653	4748
			Attitude to Health	197	522	3621
3	Red	16%	Health Care Cost *	304	636	5366
			Health Care Policy	174	659	3506
			Patient Education	173	550	3042
			Risk Factor	154	658	3452
			Cost Effectiveness Analysis	122	657	2798
			Food and Drug Administration	121	402	1720
			Prevalence	113	510	2237
4	Yellow	14%	Economics *	344	546	6333
			Socioeconomics	191	662	4303
			Public Health	167	505	2827
			Poverty	161	468	3119
			Organization and Management	132	437	2485
			Income	107	627	2499
			Health Care Delivery	93	434	2046

%: percentage of keywords that each cluster groups; (*): keyword with more occurrences and cluster name.

**Table 4:** Future research directions based on relevance score.

Future Direction of Research	Relevance Score	Main Associated Terms
Quantifying Health Systems Investment	14.033	Direct Financial Investment
		Health Care Expenditure Data
		Potential Cardiovascular Health Benefit
Green Label Education	12.965	Sustainable Consumption
		Consumer Advertising
		Sustainable Policy
Early Food Insecurity Impact	11.607	Food Insecurity Prevalence
		Yearly Health Check-up
		Culinary Medicine
WIC (Women, Infants, and Children) Nutrition Education	11.436	Educational Background
		Health Education Implication
		Individualized Telehealth Intensive Coaching
Food Waste Audit	10.231	Food Waste Impact Account
		Waste Pattern
		Cardiovascular Disease (CVD) Economic Impact
Ecological Footprint of Food	10.094	Biocultural Ecology
		Meal Cost
		Sustainable Supply Chain Management Practice
